# *NTHL1* Gene Mutations in Polish Polyposis Patients—Weighty Player or Vague Background?

**DOI:** 10.3390/ijms241914548

**Published:** 2023-09-26

**Authors:** Natalia Grot, Marta Kaczmarek-Ryś, Emilia Lis-Tanaś, Alicja Kryszczyńska, Dorota Nowakowska, Anna Jakubiuk-Tomaszuk, Jacek Paszkowski, Tomasz Banasiewicz, Szymon Hryhorowicz, Andrzej Pławski

**Affiliations:** 1 Institute of Human Genetics, Polish Academy of Sciences, Strzeszyńska 32, 60-479 Poznań, Poland; natalia.grot@igcz.poznan.pl (N.G.); marta.kaczmarek-rys@igcz.poznan.pl (M.K.-R.); emilia.lis@igcz.poznan.pl (E.L.-T.); alicja.kryszczynska@igcz.poznan.pl (A.K.); szymon.hryhorowicz@igcz.poznan.pl (S.H.); 2 Cancer Genetics Unit, Cancer Prevention Department, The Maria Sklodowska-Curie National Research Institute of Oncology in Warsaw, 02-781 Warsaw, Poland; dorota.nowakowska@coi.pl; 3 Department of Pediatric Neurology, Medical University of Bialystok, 15-089 Bialystok, Poland; anna.jakubiuk@udsk.pl; 4 Medical Genetics Unit, Mastermed Medical Center, 15-089 Bialystok, Poland; 5 Department of General and Endocrine Surgery and Gastroenterological Oncology, Poznań University of Medical Sciences, Przybyszewskiego 49, 60-355 Poznań, Poland; japaszek@op.pl (J.P.); tbanasiewicz@op.pl (T.B.)

**Keywords:** colon polyposis, CRC, FAP, *NTHL1* gene

## Abstract

Multiple polyposes are heterogeneous diseases with different underlying molecular backgrounds, sharing a common symptom: the presence of transforming into cancerous intestinal polyps. Recent reports have indicated biallelic mutations in the *NTHL1* gene, which is involved in base excision repair (BER), as predisposing to an elevated risk of colorectal cancer (CRC). We aimed to evaluate the significance of the p.Q82* truncating variant in predisposition to intestinal polyposis by assessing its frequency in polyposis patients. We genotyped 644 Polish patients and 634 control DNA samples using high-resolution melting analysis (HRM) and Sanger sequencing. We found the p.Q82* variant in four polyposis patients; in three, it was homozygous (OR = 6.90, *p* value = 0.202). Moreover, the p.R92C mutation was detected in one patient. We also looked more closely at the disease course in patients carrying *NTHL1* mutations. Two homozygous patients also presented other neoplasia. In the family case, we noticed the earlier presence of polyps in the proband and early hepatoblastoma in his brother. We cannot univocally confirm the relationship of p.Q82* with an increased risk of CRC. However, homozygous p.Q82* was more frequent by 10-fold in patients without other mutations identified, which makes *NTHL1* gene screening in this group reasonable.

## 1. Introduction

Colorectal cancer (CRC), with nearly 2 million cases per year and almost 1 million deaths, is a significant public health problem [1]. In addition, there is a marked increase in CRC incidence in both sexes [2]. In Poland, the prevalence of CRC has more than tripled in women and quadrupled in men since 1980 [3]. CRC is a heterogeneous disease with a complex etiology modulated by environmental and genetic factors [4,5]. Most CRC cases are sporadic cancers that arise by accumulating somatic point mutations in oncogenes and tumor suppressor genes [6,7]. Population studies have estimated that the hereditary form of CRC accounts for 12% to 35% of all cases [8,9]. However, it should be noted that in only 5% to 10% of all CRC cases, the disease will occur due to germline mutations in known strong predisposition genes [7,10].

**Figure 1:**
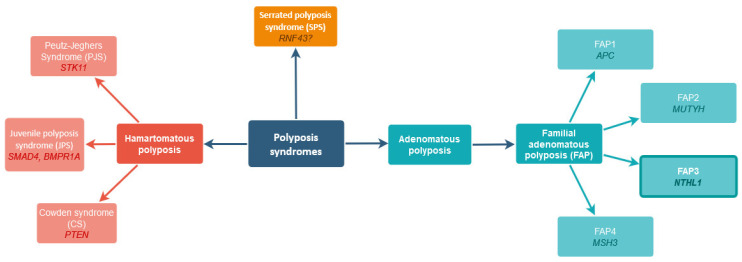
Main polyposis syndromes with genetic background.

Along with hereditary non-polypoid colorectal cancer (HNPCC), polyposis syndromes are the leading cause of hereditary CRC. Depending on the histopathological characteristics of the polyps, we can distinguish hamartomatous polyposis, adenomatous polyposis, and the rarest serrated polyposis syndrome (SPS) (Figure 1).

Hamartomatous polyposis exhibits an autosomal dominant inheritance pattern and includes Peutz-Jeghers syndrome (PJS), Juvenile polyposis syndrome (JPS), and Cowden syndrome (CS). Their main characteristic is the occurrence of hamartomatous polyposis in the gastrointestinal tract, but extraintestinal cancers are also observed. Mutations in genes responsible for the described syndromes’ development are *STK11*, *SMAD4* or *BMPR1A*, and *PTEN*, respectively [11]. SPS is a rare condition with serrated polyps sticking out from the surface of the colon or rectum, increasing the risk of developing CRC during the lifetime. The molecular basis of this disease is not fully understood, but recent studies indicate mutations in the *RNF43* gene as possibly disease-causing [12].

Although adenomas are classified as benign neoplasms, they tend to develop into carcinomas. Therefore, Familial Adenomatous Polyposis (FAP) is considered a CRC risk syndrome, with the risk of developing malignancy of up to 100% [13,14]. The OMIM classification distinguishes four types of polyposis: FAP1, which is the classic form of the disease caused by a mutation in the *APC* gene; FAP2, which comprises the disease caused by a change in the *MUTYH* gene; FAP3, where the cause of the disease is an abnormal function of *NTHL1* gene; and FAP4, which arises as a result of a mutation in the *MSH3* gene [15].

The definition of FAP3, also named *NTHL1*-associated polyposis (NAP), emerged in 2015 following Weren et al.’s report [16] of a novel polyposis syndrome associated with a homozygous substitution c.244G>A according to NM_002528.7 in the *NTHL1* gene (rs150766139, also known as c.268G>A according to NM_002528.5), which is a mutation, causing the Stop codon arising in the protein position, p.Q82* (also known as p.Q90*). Upon further investigation, mutations in the *NTHL1* gene were found to be associated not only with attenuated polyposis and colorectal cancer (CRC) but also with an increased risk of primary cancers at several different sites, including breast, meningioma, bladder, endometriosis, and basal cell carcinoma [17]. Although this alteration effect is a truncated protein, according to the ClinVar database, it is listed with the note “conflicting interpretations of pathogenicity” but, in fact, 14 records listed it as pathogenic and only one of uncertain significance.

The *NTHL1* gene is located on the short arm of chromosome 16 and encodes a protein with dual functions: DNA glycosylase and activity of DNA strand cleavage [18,19]. It belongs to the endonuclease III family, which plays an essential role in DNA repair. The best-known function of the NTHL1 protein is related to its role in repairing oxidatively induced pyrimidine lesions, thereby protecting and maintaining genomic integrity [20]. NTHL1 is one of the inducers of the base excision repair (BER) pathway, where it serves as a protein that recognizes and ultimately cuts the site of DNA damage, creating a single-strand break (SSB) [21].

Our study is part of a long-term project that deals with collecting material, detecting mutations, and determining the underlying genetic predisposition factors in cases of familial polyposis in Poland. Our ultimate goal is to enable an appropriate approach to prophylactic strategies in Polish families with polyposis, especially in those patients whose *APC*, *MUTYH*, *STK11*, *BMPR1A*, and *SMAD4* gene testing showed no abnormalities. To determine if the *NTHL1* p.Q82* mutation may be a genetic background of polyposis in Polish FAP patients with no identified mutation or if it may modify the disease course in patients with a confirmed genetic cause, we assessed the prevalence of p.Q82* in patients with familial polyposis and population control.

## 2. Results

In order to establish if the *NTHL1* p.Q82* mutation in exon 2 is associated with cancer development or influences the disease course in polyposis patients, we genotyped the gathered DNA samples using HRM analysis followed by Sanger sequencing. We assessed patients (probands) diagnosed with polyposis (*n* = 644) and healthy population controls (*n* = 634). Thus, 316 patients had no identified genetic background, and 328 were diagnosed with a mutation in one of the polyposis-causing genes.

The p.Q82* mutation was found in four unrelated patients: three were homozygous, and one was heterozygous. Moreover, the c.274C>T substitution (rs148104494, p.R92C according to NM_002528.7) was exclusively identified in one patient. In general, p.Q82* and p.R92C mutations in the *NTHL1* gene were present in 0.78% (1/129) of the whole patient group (Table 1). The mutated allele frequency was doubled compared to controls (0.62% vs. 0.32%).

In more detail, we divided the assessed patient group regarding detected mutations in other FAP-causing genes. In the group with diagnosed *APC* mutations, one patient was a heterozygous carrier of p.Q82*, and one was homozygous. Among patients with an excluded mutation in *APC* and *STK11* genes, two were homozygotes of p.Q82*, and one was a heterozygous carrier of p.R92C. That allowed us to hypothesize that homozygous p.Q82* mutations in the *NTHL1* gene increase the risk of disease seven-fold (OR = 6.92) and ten-fold regarding a group of patients without an identified genetic background (OR = 10.09). These observations were not statistically significant (*p* = 0.201 and 0.136, respectively) but noticeable, even in this relatively not numerous study group. The overall odds ratio for the mutated *NTHL1* allele was 2.52, comparing patients without identified mutation with controls, and 1.97 for the whole analyzed patient group (*p* value = 0.169 and 0.267, respectively).

**Figure 2:**
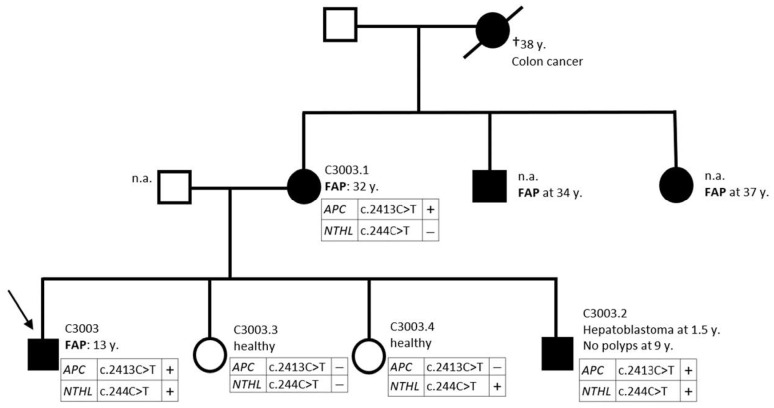
Family tree of C3003 patient. Proband was diagnosed with polyposis: *APC* c.2413C>T and *NTHL1* c.244C>T. Family interview and mutation screening of the family members was conducted, n.a.—not available.

To find the possible effects on the disease course, we focused on patients with a detected *NTHL1* mutation. Table 2 includes characteristics of patients with polyposis with p.Q82* and p.R92C mutations in the *NTHL1* gene. In one case, we also analyzed the patient’s family (Figure 2). We did not observe a straightforward dependence of disease onset or intensity. However, in the assessed family C3003, the proband, diagnosed with the *APC* c.2413C>T mutation, carrying the heterozygous p.Q82* variant, was diagnosed with FAP at the age of 13, and his affected brother at 9 still had no polyps but, in early childhood, was diagnosed with hepatoma, while the mother, who was wild type, was diagnosed with advanced disease at age 32. 

Two homozygous patients with no identified causative mutation (9434 and 9671) developed other neoplasia; in the case of the third homozygous patient (9119), we did not obtain data.

The *NTHL1* variation p.Q82* in the heterozygous state was also identified in 4 of 643 control subjects (0,63%, i.e., 1/158). 

## 3. Discussion

We found the p.Q82* mutation in the homozygous state in three polyposis patients, and in one with the detected *APC* mutation, it was heterozygous. Two patients with homozygous p.Q82* had mutations in other causative genes excluded and also developed other malignancies. In one *APC* mutation-positive patient with early onset, the p.Q82* mutation was heterozygous. Moreover, p.R92C heterozygous substitution was detected in one patient with no identified genetic background. 

Intensive studies over the past twenty years have led to the identification of genetic causes of familial polyposis, suggesting that, in addition to the *APC* gene, other predisposition genes account for a small fraction of the molecular causes of the disease. An undeniable challenge in the search for rare-risk alleles is the heterogeneity between populations, which makes it challenging to identify and validate new genes associated with familial polyposis. The prevalence and significance of the *NTHL1* gene mutations have not been described so far in the Polish population. 

Among the European population, genetic polymorphisms of the *NTHL1* gene are sporadic [22,23]. However, their role in the development of polyposis, CRC, and other cancers is supported by multiple studies [16,24,25]. 

The truncating *NTHL1* variant, p.Q82* (c.244C>T), is localized in exon 2 and is a rare alteration. It was previously described in other ethnicities [17,24,26,27], and our research revealed that it also occurs in the Polish population. In the analyzed study group, *NTHL1* p.Q82* allele T carriers were identified in 0.47% of cases with evidence of hereditary predisposition to polyposis and in 0.32% of the control group. We found no significant difference in the allele frequencies and genotype distributions of the p.Q82* mutation in the Polish patients with polyposis compared with the control group. Nonetheless, the calculated odds were ten-fold higher for homozygotes vs. other genotypes in patients with no identified mutation in FAP-related genes. Regarding group size (316), p.Q82* in the homozygous state seems to confirm earlier reports and that it is a FAP-causative alteration.

Browsing through other results, Weren et al. reported a homozygous *NTHL1* p.Q82* mutation in seven patients from three unrelated Dutch families with FAP using whole-exome sequencing, which was confirmed via Sanger sequencing. Colorectal cancer developed in four of these individuals. Other coexisting cancers that have been identified are endometrial cancer (three individuals), basal cell carcinoma (two individuals), prostate cancer (one individual), psammomatous meningioma (one individual), non-Hodgkin lymphoma (one individual), breast cancer (one individual), pancreatic cancer (one individual), duodenal cancer (one individual), and biliary tract hamartoma (one individual) [16]. In the same year, Rivera et al. described the case of a German woman suffering from multiple primary tumors, including CRC, with biallelic *NTHL1* p.Q82* mutation. Since then, the name “NTHL1 syndrome” has been proposed for this multi-cancer predisposition [24]. Also, two probands of Spanish origin [17] and two of Greek origin [26] carrying the biallelic *NTHL1* mutation have been identified. Grolleman J.E. et al. reported a homozygous *NTHL1* p.Q82* mutation in 29 patients from 17 unrelated families. Fourteen types of cancer were observed in these individuals, while almost 60% developed CRC. Interestingly, 60% of the women were diagnosed with breast cancer [25]. In the same year, cases of two patients from Denmark [28] and one from Australia [27] with a biallelic *NTHL1* p.Q82* mutation were described. We summarized the above literature data, and the p.Q82* mutation was found in the homozygous state in 44 patients before our research.

Colorectal polyps were the main symptom in patients characterized in this study, although they were histopathologically heterogeneous. Extracolonic features in case 9434 are within the range of symptoms characteristic of FAP. The number of polyps is more characteristic of attenuated polyposis. The occurrence of tumors outside the large intestine in this group was observed in two cases out of three. We do not have precise data on patient 9119. Determining the expected phenotype of carriers of biallelic *NTHL1* gene mutations would be interesting. Are these mutations always associated with intestinal polyposis, or can cancers of other organs be observed with the exclusion of bowel neoplasia? We can assume that the phenotype of breast cancer is not related to p.Q82* and mutation, which we conclude based on a study including 600 women with breast cancer where p.Q82* was not observed.

The p.R92C heterozygous substitution detected in one patient (C1167) with no identified FAP-causative mutations has conflicting interpretations of pathogenicity with ClinVar, with seven records of uncertain significance and four interpreting as likely benign, so it is difficult to resolve whether it was unequivocally causal. This patient has several adenomatous polyps identified at 60 and has no family history of malignancies. 

The *NTHL1* variation p.Q82* in the heterozygous state was also identified in 4 of 643 control subjects (0.62%, i.e., 1/158). That is more frequent than in the general European population, where according to gnomAD v2.1.1 exome analysis without filtering [29], the minor allele frequency is 0.15% (i.e., 1/647). It makes Poland more similar to Northern European populations like Sweden, where it is present with a frequency of 0.50% (according to dbSNP). What is worth pointing out is the absence of homozygotes in the population controls large cohorts, according to the databases listed above. 

Summarizing, we concluded that the *NTHL1*-related cancer predisposition in Poland occurs in 1/150,000 newborns each year. Due to this, 3–4 persons are born with *NTHL1* cancer-related risk in Poland every year, so following this line, there must be up to 400 such persons in the whole country’s population. In our research, we identified three of them. Based on the literature and our own observations, we believe that the phenotype of homozygous NTHL1 gene mutations is not a classic case of FAP. In studies, Grolleman H.E. et al. concluded that 60% of 29 his p.Q82* homozygous patients developed CRC. Additionally, he observed the incidence of BC in 60% of women in this group [25]. In our homozygous carriers group, two were females with no diagnosed breast cancer. Additionally, we did not observe homozygous carriers in 600 women with breast cancer. The phenotype of most expected p.Q82* homozygous carriers in the Polish population remains undetermined.

All these facts make the p.Q82* of the *NTHL1* gene screening reasonable in polyposis patients (especially in an unusual disease course), with *APC*, *MUTYH*, *STK11*, *BMPR1A*, and *SMAD4* mutations excluded.

## 4. Materials and Methods

### 4.1. Patients

The study included a group of 644 patients (320 males and 324 females) with intestinal polyposis from Poland. The diagnosis of FAP in the tested group relied on family history collection, analysis of clinical symptoms, and endoscopy or complete colonoscopy. The subjects were divided into two groups: patients with previously diagnosed FAP-related mutations (328 individuals) and sporadic cases with excluded mutations in the genes mentioned above (316 individuals). The control group was recruited among healthy patients and included 634 subjects (317 males and 317 females). The patients enrolled in the present study declared Polish descent. All tested individuals provided written informed consent to participate in genetic testing approved by the local ethical committee (Ethics Committee of the Poznan University of Medical Sciences (approval no. 459/10)).

### 4.2. Genetic Analyses

Genomic DNA was isolated from peripheral blood leukocytes using guanidine isothiocyanate and phenol-chloroform extraction following the standard protocol [30]. Isolates were dissolved in 1xAE buffer (Tris-EDTA, 10 mM; pH = 9), diluted to a 50 ng/ul concentration, and stored at 4 °C until use.

The genotyping of the *NTHL1* variants was performed via high-resolution melting analysis (HRM). The HRM was carried out using the Rotor-Gene Q thermocycler (Qiagen, Hilden, Germany) and performed in a final volume of 16 μL. The reaction mixture contained 3 μL 5× Hot FIREPol EvaGreen HRM Mix (Solis BioDyne, Tartu, Estonia), 50 ng of DNA template, 5.5 pmol/μL of NTHL1_F and NTHL1_R primers, and 9.8 μL of PCR-grade water. The DNA fragments were amplified using real-time PCR by an initial denaturation at 95 °C for 5 min and then 40 cycles as follows: 95 °C for 10 s, 60 °C for 30 s, and 72 °C for 10 s. In order to determine the melting points, a melting curve analysis was performed by slowly raising the temperature from 65 °C to 95 °C by 0.1 °C at each step. The melting was further analyzed using the Rotor-Gene Q Series software version 2.3.4 (Qiagen). We included heterozygote control, positive for the *NTHL1* p.Q82* variant, to provide detection of the specific mutation (Figure S1). The detected variants were verified with Sanger sequencing using the commercial BigDye™ Terminator v3.1 Cycle Sequencing Kit (Applied Biosystems) (Figures S2 and S3). *NTHL1* sequence variants in the heterozygous and homozygous state were aligned with the wild type using MEGA X Software (Figure S4). Primer3Plus Online Software was applied to design the sequences of the primers used in the analysis. The primer sequences were as follows: forward (NTHL1_F) 5′-AGAGACTGCGTGTGGCCTA and reverse (NTHL1_R) 5′-GAGGGTGCCAGCCAAAAG, and the amplicon size was 284 bp.

### 4.3. Statistical Analysis

The frequency of *NTHL1* p.Q82* and mutation among patients compared to healthy controls was presented as a percentage frequency distribution. The odds ratios (ORs) with 95% confidence intervals (95% CIs) and *p* values were also calculated to compare groups. Hardy–Weinberg equilibrium (HWE) was analyzed by using the χ^2^ test. MedCalc Software Version 22.009 was used for statistical calculations [31]. As an indicator of statistical significance, we considered *p* values less than 0.05.

## 5. Conclusions

We examined 644 polyposis probands and 634 control subjects from the Polish population for the presence of p.Q82* in the NTHL1 gene. Three homozygous carriers and one FAP family with heterozygous p.Q82* were identified in the polyposis patient group. Four heterozygous carriers were identified in the control group. The prevalence of p.Q82* in the Polish population is 1 per 158, and 300–400 homozygous carriers of p.Q82* can be expected in Poland. The p.Q82* homozygous polyposis patients do not have classic FAP features. The polyps were not numerous and histopathological; they were hyperplastic or serrated. In these cases, other symptoms outside of the gut were observed. Heterozygous NTHL1 p.Q82* is not associated with an increased risk of CRC in the Polish population and does not modify the FAP course. However, our results indicate that in the homozygous state, it is associated with a 10-fold higher risk of CRC in polyposis patients without identified mutations in polyposis-related genes. Further studies involving other types of cancers and other populations are needed to determine the exact role of this variant. In patients with polyposis who do not have a mutation that causes polyposis, screening for the NTHL1 gene in the p.Q82* variant should be recommended, but in the case of an atypical course of FAP, APAP, or hamartomatous and serrated polyposis syndromes only.

## Figures and Tables

**Table 1:** Distribution of genotype and allele frequencies of *NTHL1* gene in patients with polyposis and healthy controls with their statistical significance. Mutated alleles (p.Q82* and p.R92C) were summarized and tagged as X and wild-type alleles as x.

*NTHL1* Mutation	Genotypes *n* (%)			Alleles *n* (%)	
Group	xx	Xx	XX	x	X
Polyposis patients whole group (*n* = 644)	639 (99.22%)	2 (0.31%)	3 (0.47%)	1280 (99.38%)	8 (0.62%)
Polyposis patients (no identified genetic background) * (*n* = 316)	313 (99.05%)	1 (0.32%)	2 (0.63%)	627 (99.21%)	5 (0.79%)
Polyposis patients with an identified mutation (*n* = 328)	326 (99.39%)	1 (0.305%)	1 (0.305%)	653 (99.54%)	3 (0.46%)
Controls (*n* = 634)	630 (99.37%)	4 (0.63%)	n.o.	1264 (99.68%)	4 (0.32%)
	[XX] vs. [xx + Xx]	[XX + Xx] vs. [xx]	[XX] vs. [xx]	[x] vs. [X]	[X] vs. [x]
Polyposis patients (whole group) vs. controls *p* value OR (95% CI)	OR = 6.92 CI = [0.36–134.32] *p* value = 0.201	OR = 1.23 CI = [0.33–4.61] *p* value = 0.756	OR = 6.90 CI = [0.36–133.89] *p* value = 0.202	OR = 0.45 CI = [0.14–1.46]	OR = 1.97 CI = [ 0.59–6.58]
				*p* value = 0.267	
Polyposis patients (no identified genetic background) vs. controls *p* value OR (95% CI)	OR = 10.09 CI = [0.48–210.76] *p* value = 0.136	OR = 1.51 CI = [0.34–6.79] *p* value = 0.591	OR = 10.06 CI = [0.48–210.10] *p* value = 0.137	OR = 0.40 CI = [0.11–1.48]	OR = 2.52 CI = [ 0.67–9.42]
				*p* value = 0.169	
Polyposis patients with an identified mutation vs. controls *p* value OR (95% CI)	OR = 5.81 CI = [0.24–143.08] *p* value = 0.282	OR = 0.97 CI = [0.18–5.30] *p* value = 0.969	OR = 5.79 CI = [0.24–142.62] *p* value = 0.283	OR = 0.69 CI = [0.15–3.09]	OR = 1.45 CI = [0.32–6.51]
				*p* value = 0.626	

n.o.—not observed; X—mutated allele, x—wild type allele; [XX] vs. [xx + Xx]—dominant model; [XX + Xx] vs. [xx]—recessive model. No identified genetic background *—mutations in *APC*, *MUTYH*, *STK11*, *BMPR1A*, and *SMAD4* excluded.

**Table 2:** Characteristics of patients with polyposis with p.Q82* and p.R92C mutations in the *NTHL1* gene.

Family	Patient ID	*NTHL1* Variant	Allelic State	Amino Acid Change	Malignancies	Polyps	Co-Occurrent Mutation
1	9119	c.244C>T	homozygous	p.Q82*	no accessible data	adenomatous	APC c.4129_4130delGT
2	9434	c.244C>T	homozygous	p.Q82*	papillary thyroid cancer at 60	tubular, hyperplastic, serrated, above 100	n.d.
3	9671	c.244C>T	homozygous	p.Q82*	occipital meningioma, two meningiomas of the left frontal lobe diagnosed at the age of 69; at 43, hysterectomy due to fibroids and heavy bleeding	histopathologically villous and tubulovillous adenomas at the age of 61	n.d.
4	C1167	c.274C>T	heterozygous	p.R92C	no family history	dozen adenomatous polyps at 60	n.d.
5	C3003	c.244C>T	heterozygous	p.Q82*		adenomatous at 13	APC c.2413C>T
	C3003.1	c.244C>T	-	-		adenomatous at 32	APC c.2413C>T
	C3003.2	c.244C>T	heterozygous	p.Q82*	hepatoblastoma at 1.5	no polyps at 9	APC c.2413C>T
	C3003.3	n.d.	-	-		healthy	APC c.2413C>T excluded
	C3003.4	c.244C>T	heterozygous	p.Q82*		healthy	APC c.2413C>T excluded

n.d.—not detected.

## Data Availability

The data presented in this study are available on request from the corresponding author.

## Supplementary Materials

The following supporting information can be downloaded at: https://www.mdpi.com/article/10.3390/ijms241914548/s1.
