# Supplementary material for: NTHL1 Gene Mutations in Polish Polyposis Patients—Weighty Player or Vague Background?
Source: Int J Mol Sci. 2023 Sep 26;24(19):14548. doi: 10.3390/ijms241914548 (PMC10572874; doi:10.3390/ijms241914548)
Supplement: Supplementary file 1 [file ijms-24-14548-s001.zip › ijms-2601159-supplementary.pdf]

Supplementary Materials

# *NTHL1* gene mutations in Polish polyposis patients - weighty player or vague background?

**Natalia Grot[1], Marta Kaczmarek-Ryś[1], Emila Lis-Tanaś[1], Alicja Kryszczyńska[1], Dorota Nowakowska[2], Anna Jakubiuk-Tomaszuk[3,4], Jacek Paszkowski[5], Tomasz Banasiewicz[5], Szymon Hryhorowicz[1] and Andrzej Pławski[1,5] ***

[1] Institute of Human Genetics, Polish Academy of Sciences, Strzeszyńska 32, 60-479 Poznań, Poland; natalia.grot@igcz.poznan.pl (N.G.); marta.kaczmarek-rys@igcz.poznan.pl (M.K.-R.); szymon.hryhorowicz@igcz.poznan.pl (S.H.); emilia.lis@igcz.poznan.pl (E.L.-T.); alicja.kryszczynska@igcz.poznan.pl (A.K.); andp@man.poznan.pl (A.P.)

[2] Cancer Genetics Unit, Cancer Prevention Department, The Maria Sklodowska-Curie National Research Institute of Oncology in Warsaw, Warsaw, Poland, dorota.nowakowska@coi.pl (D.N.) ORCID: 0000-0002-7109-5284

[3] Department of Pediatric Neurology, Medical University of Bialystok, Bialystok, Poland, anna.jakubiuk@udsk.pl (A. J. T.)

[4] Medical Genetics Unit, Mastermed Medical Center, Bialystok, Poland, ajaktom@gmail.com (A. J. T.)

[5] Department of General and Endocrine Surgery and Gastroenterological Oncology, Poznań University of Medical Sciences, Przybyszewskiego 49, 60-355 Poznań, Poland; japaszek@op.pl (J.P.); tbanasiewicz@op.pl (T.B.)

* Correspondence: andp@man.poznan.pl; Tel.: +48(60)4547635

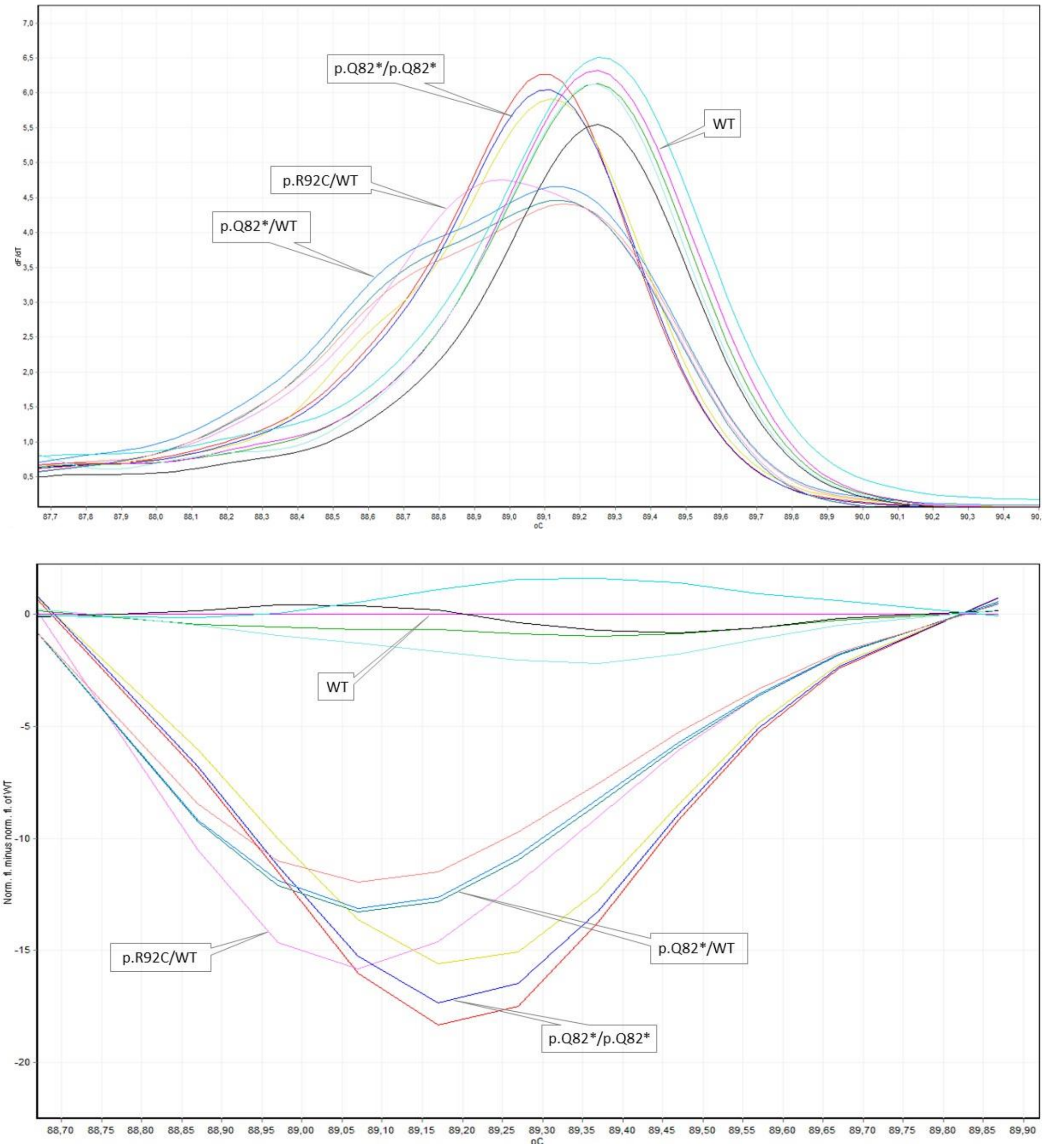


**Figure S1.** Melting profiles from HRM analysis indicating *NTHL1* mutations in exon 2.

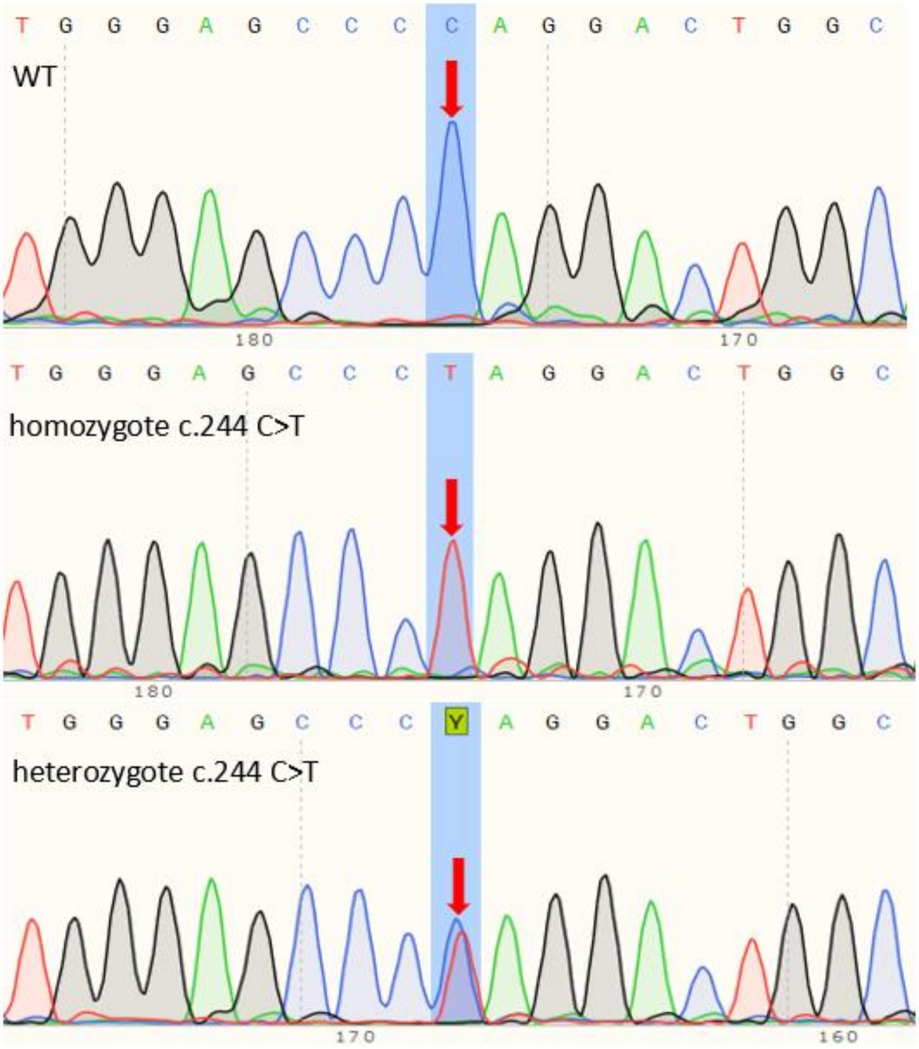


**Figure S2.** Sequencing chromatograms demonstrating c.244 C>T mutations in the homozygous and heterozygous state compared to wild type in the *NTHL1* gene.

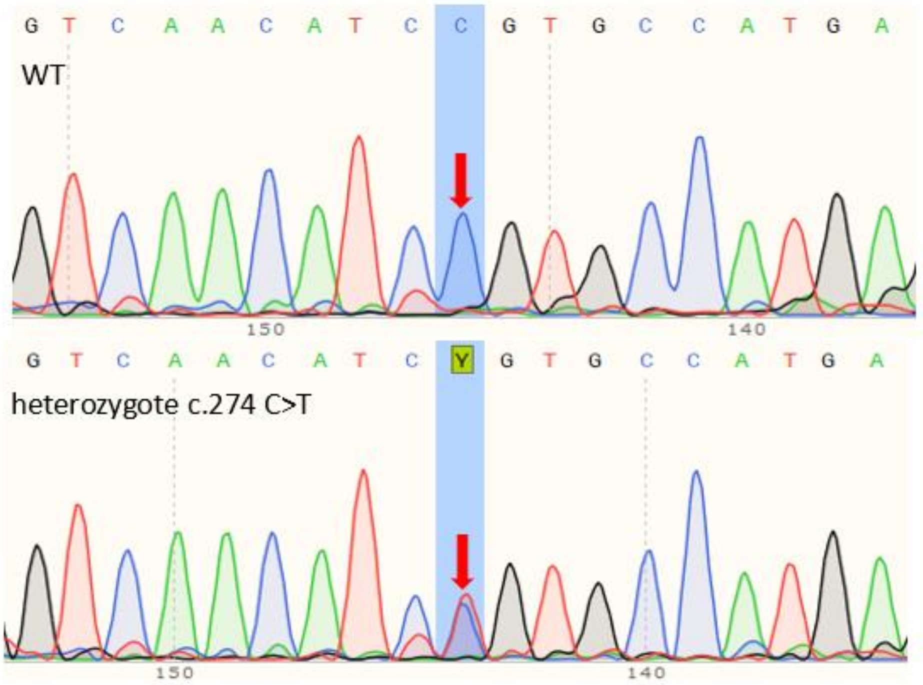


**Figure S3.** Sequencing chromatograms demonstrating c.274 C>T mutation in the heterozygous state compared to wild type in the *NTHL1* gene.

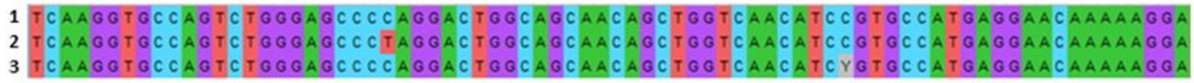


**Figure S4.** Result of multiple sequence alignment of *NTHL1* variants.
1 - WT; 2 - homozygote c.244 C>T; 3 - heterozygote c.274 C>T.
